# Spontaneous Spinal Epidural Hematoma: A Case of a Benign Presentation and Emergency Department Management

**DOI:** 10.7759/cureus.23532

**Published:** 2022-03-27

**Authors:** Derrick Huang, Stephanie Iken, Samyr Elbadri, Michael Falgiani, Latha Ganti

**Affiliations:** 1 Emergency Medicine, HCA Florida Ocala Hospital, Ocala, USA; 2 Emergency Medicine, University of Central Florida College of Medicine, Orlando, USA; 3 Emergency Medicine, Envision Physician Services, Plantation, USA; 4 Emergency Medicine, Hospital Corporation of America (HCA) Florida Ocala Hospital, Ocala, USA

**Keywords:** spontaneous intracerebral hemorrhage, extradural bleeding, neurologic emergency, spinal epidural hematoma (seh), spontaneous spinal epidural hematoma

## Abstract

Spontaneous spinal epidural hematomas (SSEHs) are neurological emergencies complicated by a wide array of presentations. In this study, we report a case of a patient who presented with neck pain and was diagnosed with an SSEH with computed tomography (CT) angiography with subsequent confirmation by magnetic resonance imaging (MRI). The high-risk location and size of the lesion guided management and surgical intervention. In a stable patient presenting to the emergency department without focal neurological deficits, clinical suspicion and assessment of risk factors are integral in the evaluation of patient risk and subsequent imaging and intervention.

## Introduction

Spontaneous spinal epidural hematomas (SSEHs) are both a neurological emergency and a rare emergency department (ED) presentation, with an estimated frequency of 0.1 cases per 100,000 population annually and increasing incidences with greater use of spinal magnetic resonance imaging (MRI) [1,2]. Spinal epidural hematomas typically occur from extradural bleeding with an inciting element on history, such as trauma, epidural anesthesia, or a complication of an operative intervention [1]. However, SSEHs occur without known instigation and are complicated by a wide array of presentations based on the degree and site of spinal cord compression, ranging from nonspecific back pain with or without radiculopathy to hemiparesis, urinary retention, and spinal shock with high cervical involvement [3-6]. Furthermore, there can be delays from the onset of back pain to neurological symptoms, with cases reported to range from hours to several days or even months from the onset of the back pain [7]. Without an accompanying history of trauma, these neurological symptoms can overlap with cerebral vascular accidents (CVAs) and transient ischemic attacks (TIAs) that may increase the likelihood of mismanagement [1,3-5]. Crucially, the prognosis of SSEH is time-sensitive and related to the severity of symptoms on presentation. Delays in diagnosis may result in permanent neurological sequelae and death [1,3].

In the setting of atraumatic neck and back pain, primary ED management is based on the findings of “red flags” that differentiate musculoskeletal pathology from life-threatening processes such as immunosuppression and fever suggesting infection, anterior localization suggesting cardiac or esophageal etiology, and neurological symptoms that may necessitate time-sensitive treatment and imaging for CVAs [8]. In the case of a benign presentation, a thorough clinical exam assessing for disease progression, risk factors, and neurological symptoms is essential. We present a young adult patient who presented with neck pain and was diagnosed with an SSEH after computed tomography angiography (CTA) and subsequent MRI confirmation.

## Case presentation

A 37-year-old male without past medical history presented with neck pain. The patient stated that he initially woke up with neck soreness three days ago. The pain worsened later that day, prompting the patient to go to an ED where he was discharged with a muscle relaxant after a negative computed tomography (CT) scan. The patient described the pain as stabbing in the midline of his back, waxing, and waning, occasionally shooting into his shoulders bilaterally. He experienced occasional numbness and tingling from his humerus down to his arms, more significant on his right side. He denied extremity weakness or a history of trauma or specific injury prior to the pain. He had no urinary or bowel incontinence. The patient stated he does lift weights and is employed at a manufacturing plant lifting heavy pipes, but he stated that he had not performed any heavy lifting recently. He denied fevers, chest pain, difficulty breathing, and any history of herbal medicine, blood thinner use, spinal surgery, cancer, or intravenous drug use. The patient stated that he used anabolic steroids.

On his initial vitals, the patient had pulse oximetry of 98% on room air, blood pressure of 159/90 mmHg, temperature of 36.4°C, heart rate of 70 beats per minute, and a respiratory rate of 18 breaths per minute. On physical examination, the patient was alert and oriented without distress. The patient had midline cervical to upper thoracic spine tenderness to palpation. He had bilateral trapezius muscle spasms and tenderness to palpation. He had a full range of motion of his neck without worsening paresthesia on neck flexion. The upper and lower extremities were without motor or sensory deficit. The patient otherwise had a regular heart rate and rhythm, equal pulses bilaterally, and lungs were clear to auscultation. The abdomen was soft and non-tender. The skin exam was unremarkable.

**Figure 1:**
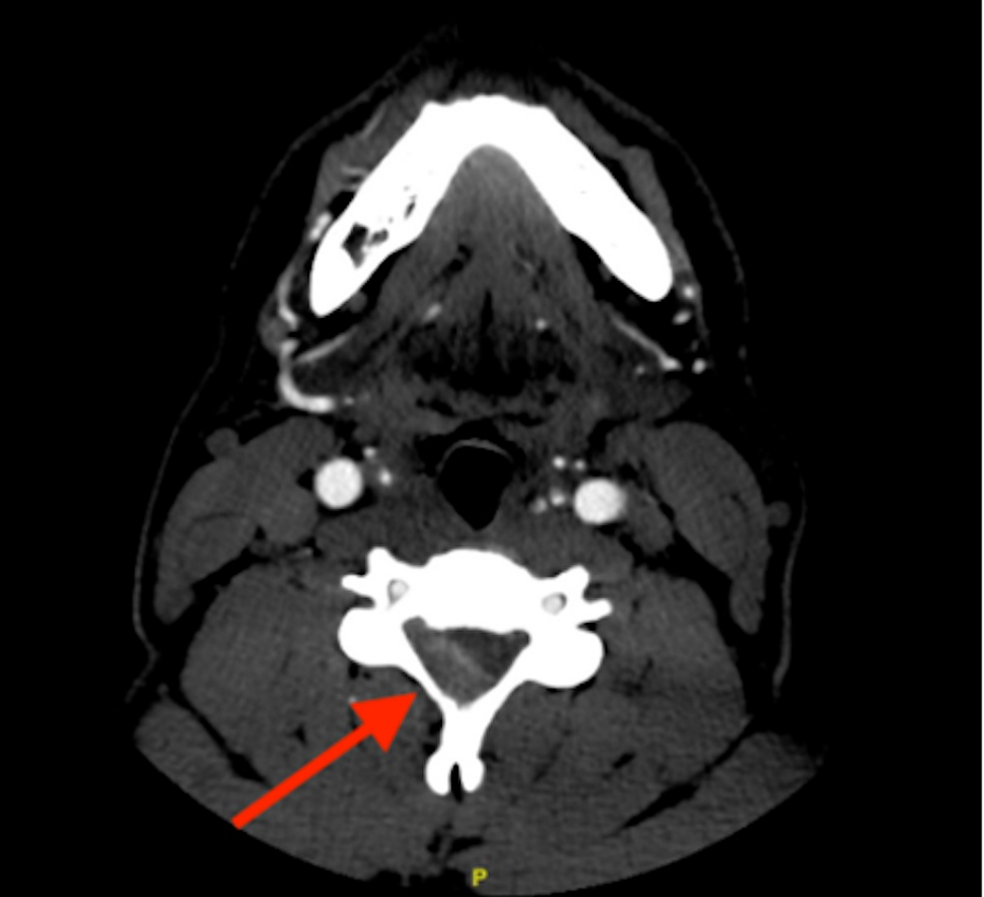
Axial CT with angiography image at the C3 level showing a right-sided epidural hematoma with an associated sac and cord compression in a left anterolateral direction

Initial laboratory studies showed hemoglobin, hematocrit, and white blood cell count within normal ranges. Electrolytes and coagulation profiles were also unremarkable. CT head and cervical spine without contrast were unremarkable. CTA of the neck showed normal cervical carotid arteries. CTA of the head showed right posterolateral epidural density representing a right-sided epidural hematoma at the C3-C7 level with an associated sac and cord compression in a left anterolateral direction (Figure 1).

**Figure 2:**
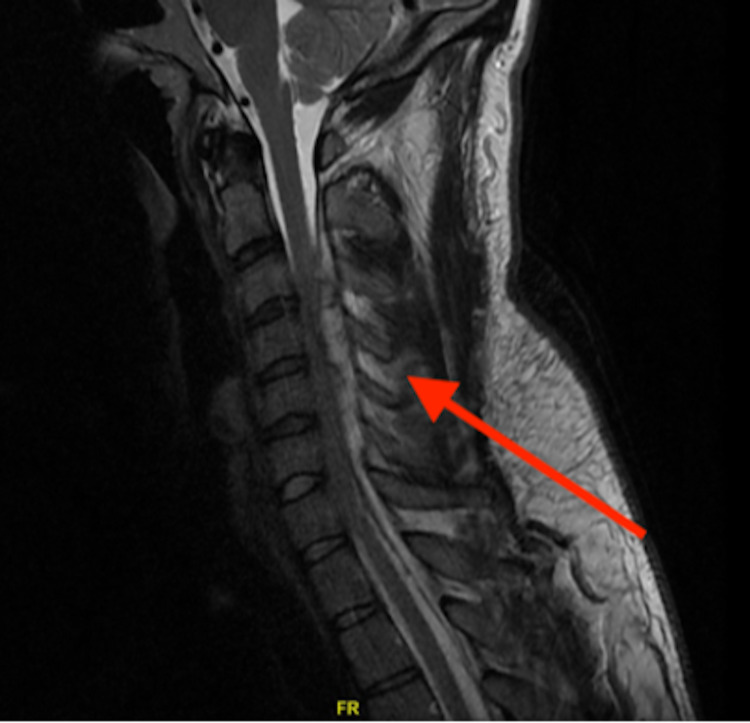
Sagittal T2 enhanced cervical MRI showing posterior epidural heterogenous collection representing an epidural hematoma extending from the C2-3 level to the T3 level with moderate flattening of the cord

Findings prompted an order for 10 mg dexamethasone IV and enhanced MRI imaging, which showed a large posterior epidural heterogenous collection representing an epidural hematoma extending from the C2-3 level to the T3 level with moderate flattening of the cord (Figure 2).

**Figure 3:**
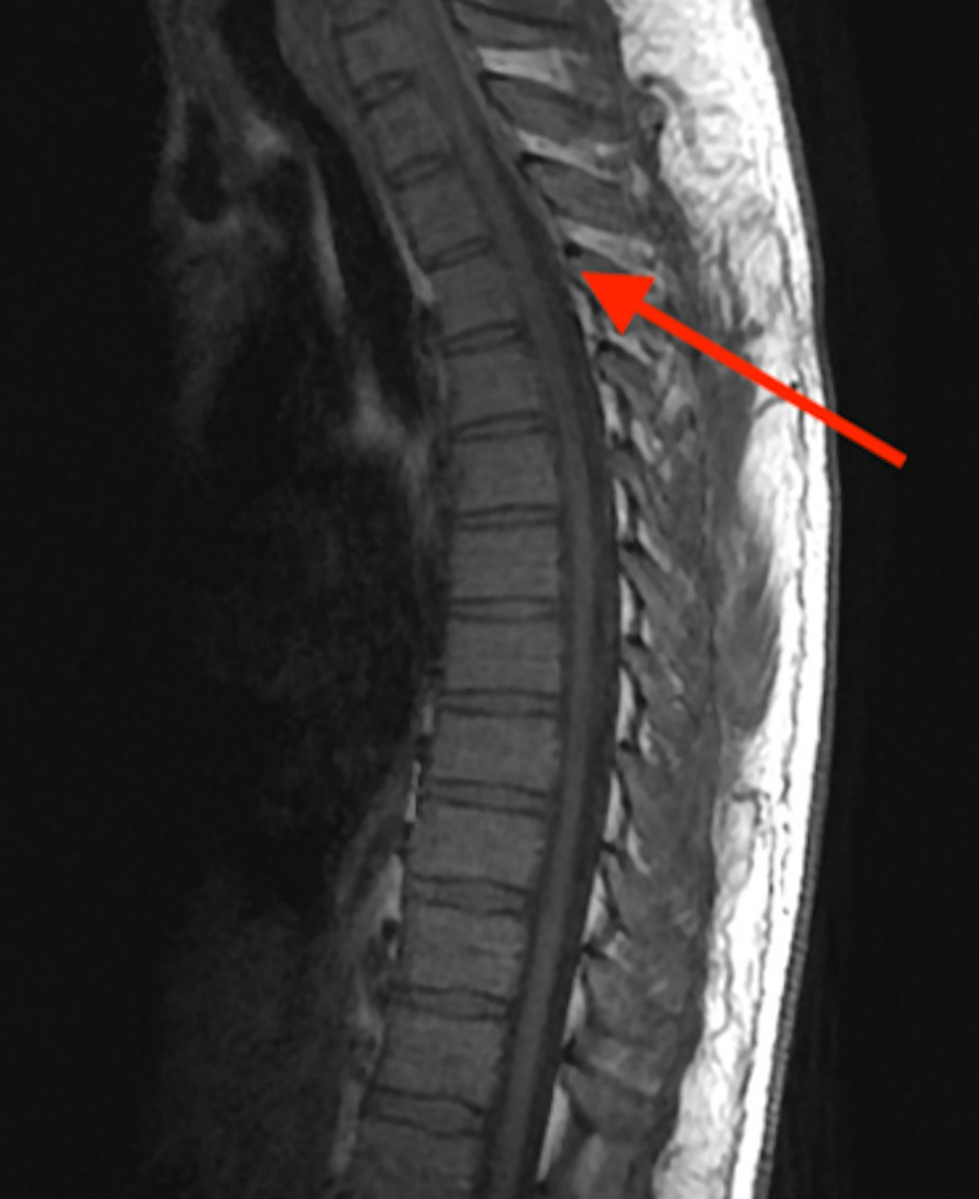
Sagittal T2 enhanced thoracic MRI showing severe spinal canal stenosis at T1 and moderate spinal canal stenosis at T2

Cervical epidural hematoma extended ventrally to the T2-3 level and dorsally to the T4-5 level with severe spinal canal stenosis at T1 and moderate spinal canal stenosis at T2 (Figure 3). The patient was admitted to the intensive care unit (ICU) for neurological monitoring and proceeded to have an uncomplicated posterior cervical laminectomy and fusion the next day.

## Discussion

We report a case of SSEH in a patient without focal neurological deficits. Our patient was first assessed for red-flag symptoms suggesting life-threatening pathology. Given his young age, absence of cardiac risk factors, and intermittent, bilaterality of symptoms, he was unlikely to have had a TIA or stroke, which would have necessitated a non-emergent TIA work-up or thrombolysis in a patient with a potentially enlarging epidural hematoma [4,5].

Due to our patient’s significant history of heavy lifting, in both recreational and occupational settings with reported anabolic steroid use, and continued neurologic symptoms despite previous outpatient treatment, the patient was deemed to be at a higher risk and underwent CTA and ultimately a confirmatory MRI study. Although MRI is the study of choice for the diagnosis of SSEH, CT imaging has been suggested if MRI is not immediately available, as is the case in many hospital settings [3,9]. Although CTA may not be sufficiently sensitive for all SSEHs, the use of CTA may be of additional benefit due to its potential to rapidly monitor worsening pathology, screen for larger hematomas, and simultaneous assessment of CVA etiologies, given the potentially similar symptomology between SSEH and CVA/TIAs [10]. Although no studies have assessed the effectiveness of CTA as an initial imaging tool, in our case, CTA was able to screen for an epidural bleed, allowing for subsequent MRI confirmation (Figure 1). Future studies are required to assess for further use of angiography to facilitate risk stratification, particularly in stable patients without focal neurological deficits. Dexamethasone was also promptly administered, given that the pathophysiology of SSEH involves direct compression or vascular compression, causing progressive spinal cord edema and ischemia [11]. In metastatic spinal cord compression, when systemic side effects such as infection are carefully considered, dexamethasone is the adjunctive therapy of choice, given that the treatment decreases tissue edema and inflammation at the site of cord compression, resulting in symptomatic relief and potentially facilitating recovery [7,11-13].

SSEHs have been reported in a wide age range with several risk factors that may facilitate diagnosis. These risk factors include the use of anticoagulation or antiplatelet agents, such as warfarin, direct oral anticoagulants, and aspirin; those predisposed to bleeding due to genetic diseases, such as hemophilia and von Willebrand disease; and those using certain herbal supplements [1,14,15]. Pregnancy and exertion (i.e., Valsalva maneuver) have also been described in younger patients presenting with SSEH [1,9,16]. Of particular importance in our patient was his history of heavy lifting in both occupational and recreational settings. Prior cases of SSEH strongly suggest that the venous system is the most likely source of bleeding. The posterior epidural venous plexus does not contain valves, and thus, it is at the risk of increased venous pressure transmission and rupture from intrathoracic and intraabdominal compartments on straining [3,16]. Additionally, there is a higher reported prevalence of cervical and cervicothoracic lesions possibly due to this spinal region possessing a compact continuous venous network in the epidural space [1,3]. Although our patient presented with an initially elevated blood pressure and may have had pre-existing hypertension, hypertension has not been shown to be a risk factor based on existing reports [13].

In the ED, providers should be aware that the management of SSEH is highly time-sensitive, and there is a potential for rapid deterioration [1,3,7,17]. Although studies have not proven a statistically significant difference in the outcomes based on the time from symptom onset to surgery and as no randomized controlled trials (RCTs) have been performed on surgical versus nonsurgical outcomes in cases without severe neurological deficits, experts continue to suggest that patients should undergo surgical decompression within 12 to 48 hours of symptom onset for optimal outcomes, given the potential for continued and rapid neurological deterioration [1,7,18]. Although our patient had a benign ED presentation, he had greater than four spinal segments of involvement and thoracic spine involvement, where there is less space available for the cord to tolerate hematoma expansion, both predictors for poor outcome [17,18]. Given these findings and the potential for rapid neurological deterioration, our patient’s disposition was immediate to the ICU for close monitoring and ultimately to surgical decompression for definitive management.

## Conclusions

Despite limited definitive evidence-based management due to the paucity of studies on SSEHs, numerous reports provide an insight into potential management pathways. Thorough clinical examination, including assessment of risk factors and a diligent differential diagnosis, is essential in the assessment of SSEHs. CTA imaging and knowledge of high-risk lesions found on MRI may be beneficial in monitoring and disposition.
